# Herbal Extracts of Ginseng and Maqui Berry Show Only Minimal Effects on an In Vitro Model of Early Fracture Repair of Smokers

**DOI:** 10.3390/foods12152960

**Published:** 2023-08-04

**Authors:** Helen Rinderknecht, Alana Mayer, Tina Histing, Sabrina Ehnert, Andreas Nüssler

**Affiliations:** Siegfried-Weller Institute for Trauma Research, BG Trauma Center, University of Tuebingen, Schnarrenbergstrasse 95, 72070 Tuebingen, Germany; helen.rinderknecht@student.uni-tuebingen.de (H.R.); alana.mayer@student.reutlingen-university.de (A.M.); thisting@bgu-tuebingen.de (T.H.); sabrina.ehnert@med.uni-tuebingen.de (S.E.)

**Keywords:** *Panax ginseng*, maqui berry, trauma, bone, fracture repair, smoking, in vitro, angiogenesis

## Abstract

Smoking is a major risk factor for delayed fracture healing, affecting several aspects of early fracture repair, including inflammation, osteogenesis, and angiogenesis. *Panax ginseng* (GE) and maqui berry extract (MBE) were shown in our previous studies to reduce smoke-induced cellular damage in late bone-healing in vitro models. We aimed here to analyze their effects on the early fracture repair of smokers in a 3D co-culture model of fracture hematomas and endothelial cells. Both extracts did not alter the cellular viability at concentrations of up to 100 µg/mL. In early fracture repair in vitro, they were unable to reduce smoking-induced inflammation and induce osteo- or chondrogenicity. Regarding angiogenesis, smoking-induced stress in HUVECs could not be counteracted by both extracts. Furthermore, smoking-impaired tube formation was not restored by GE but was harmed by MBE. However, GE promoted angiogenesis initiation under smoking conditions via the *Angpt/Tie2* axis. To summarize, cigarette smoking strikingly affected early fracture healing processes in vitro, but herbal extracts at the applied doses had only a limited effect. Since both extracts were shown before to be very effective in later stages of fracture healing, our data suggest that their early use immediately after fracture does not appear to negatively impact later beneficial effects.

## 1. Introduction

Among the most common injuries treated in emergency rooms worldwide are bone fractures. In Germany, in the year 2019, the fracture incidence was around 1 per 100 inhabitants, which is a 14% increase in comparison to the year 2009 [1]. Between 5 and 10% of all fractures show a delay in healing or even result in non-unions, and several risk factors such as, for instance, age and gender, medication, or comorbidities like diabetes mellitus are known to delay the healing process [2]. One of the main underrated risk factors is the consumption of cigarettes. Even though the health risks of smoking are widely known, in Germany, around 35% of adults consume cigarettes daily. Smoking is therefore one of the greatest preventable health risks [3,4].

Fracture healing is an overly complex process due to the interplay of the immune system, osteogenesis, and angiogenesis [5]. Fracture repair begins directly after the fracturing of the bone with the formation of a fracture hematoma in the fracture gap, which is formed due to the rupture of the nearby vasculature, resulting in a hypoxic environment [6]. The fracture hematoma stabilizes the fracture ends, and initiates and orchestrates the inflammatory phase of healing. The early inflammatory phase is determined by the specific recruitment of inflammatory and immune cells guided by the secretion of specific cytokines and growth factors, including interleukin (IL)-1β, IL-8, IL-6, tumor necrosis Factor-alpha (TNF-α), C-C motif chemokine-2 (CCL2), transforming growth factor-beta (TGF-β), platelet-derived growth factor (PDGF), fibroblast growth factor (FGF), or vascular endothelial growth factor (VEGF), by both resident and invading cells [5,7]. The recruitment and proliferation of osteoprogenitors support healing, while suppressing inflammation. Fracture hematomas are dissolved by macrophages within several days after fracture. The differentiation of the stem cells, hypertrophic chondrocytes, and other bone cells leads to callus formation and remodeling and, finally, the rebuilding of the bony structure via intramembranous bone formation [8].

Inadequate vasculature is one of the main reasons for impaired bone and wound healing, but detailed information on revascularization in the early stages of fracture repair is still limited [9,10]. During fracture repair, vessels are mainly formed by angiogenesis, tightly coupled with osteogenesis [11,12]. Due to capping from the blood supply, hypoxia in the fracture environment leads to the secretion of VEGF from adjacent chondrocytes and infiltrating macrophages, stimulating endothelial proliferation, differentiation, and vascular permeability [13]. Besides VEGF, other growth factors such as platelet-induced growth factors (PIGFs), FGFs, PDGFs, IGFs, or angiopoietins (Angpts) participate in the angiogenesis of bone fractures [14].

Smoking may affect fracture repair at different levels. Smokers are known to have an altered immune cell composition and activity, as well as a general hyperinflammatory state [15]. Furthermore, they exhibit reduced bone mineral densities, accompanied by an increased risk of fracture and impaired mesenchymal stem cells (MSCs) [16,17]. Lastly, smoking also affects the vasculature and has been widely associated with endothelial dysfunction and cardiovascular diseases [18]. Both in vitro and in vivo, it could be shown that early fracture repair under smoking conditions is characterized by a hyper-inflammatory state, reduced osteogenic differentiation, and strongly dysregulated and impaired angiogenesis [19,20,21]. In addition, dysregulation of the Angpt/Tie2 axis was found to supposedly impair early angiogenic events in smokers [20].

Herbal extracts, often associated with antioxidant properties, have been widely used in traditional medicines worldwide. In Western medicine, herbal extracts are not one of the first choices, even though they rarely have severe side effects [22]. In previous studies, we could show that herbal extracts of *Panax ginseng* roots and maqui berries reduce smoking-induced damage in osteoblasts and a bone co-culture model of late fracture healing [23,24]. Especially known in traditional Asian medicine, extracts from *P. ginseng* roots (GEs) have been linked to anti-inflammatory, anti-oxidative, as well as pro-osteogenic and angiogenic effects in vitro and in vivo, thereby also assisting in fracture repair [25,26,27,28,29]. Their biologically active components include saponins, flavonoids, and polysaccharides. Most abundant are saponins, which include ginseng-specific ginsenosides, making up approximately 30% of all saponins. The most abundant ginsenosides are Rg1, Rb1, Rd, and R1 [30]. Extracts from maqui berries (MBEs), *Aristotelia chilensis*, exotic fruits domestic to Chile, are rich in polyphenols and anthocyanins, including delphinidin [31]. The extracts have a very high antioxidant capacity and also exhibit anti-inflammatory effects [32]. Moreover, anthocyanins and delphinidins have been shown to promote bone mineralization and osteoblastic differentiation and to inhibit osteoclastogenesis [33,34]. In contrast to GE, MBE, and anthocyanins are generally considered to have an anti-angiogenic effect [35,36].

In this study, we aimed to analyze the effects of GE and MBE on the early impaired fracture healing of smokers in vitro, as the extracts have previously been shown to reduce smoking-induced damages in bone culture models [23,24]. Due to the importance of the interplay between the vasculature and the bony tissue, an analysis was performed using an in vitro co-culture approach, combining our previously established in vitro fracture hematomas and endothelial cells [20]. As hallmarks of early fracture repair, in vitro models were analyzed with regard to inflammation, osteogenic and chondrogenic differentiation potential, and angiogenesis.

## 2. Materials and Methods

### 2.1. Cell Lines and Culture

As representative of mesenchymal stem cells, the human immortalized stem cell line SCP-1 was used [37]. SCP-1 cells were cultured in Minimal Essential Medium Alpha (MEM-α, HiMedia Laboratories, Thane West, Maharashtra, India), supplemented with 5% fetal calf serum (FCS, Thermo Fisher, Waltham, MA, USA). Human umbilical cord vein cells (HUVEC) were used as a proxy for endothelial cells. HUVECs were cultured in Endothelial Cell Growth Basal Medium 2 (EBM-2, Peprotech, Hamburg, Germany), supplemented with 2% FCS, 1% Antibiotic/Antimycotic (A/A, PAA Laboratories, Toronto, Canada), 0.5 ng/mL human VEGFA165, 10 ng/mL human FGF-b, 5 ng/mL human epidermal growth factor (EGF), 20 ng/mL human IGF-R3 (all Peprotech, Hamburg, Germany), 22.5 µg/mL heparin (Leo Pharma, Bellerup, Denmark), 0.2 µg/mL hydrocortisone (Pfizer, New York, NY, USA), and 1 µg/mL L-ascorbic acid (Sigma-Aldrich/Merck, Darmstadt, Germany) in culture flasks coated with 0.1% gelatin (Sigma-Aldrich/Merck, Darmstadt, Germany). The culture medium was replaced every 3–4 days. The cells were subcultured upon reaching confluency. SCP-1 cells in passages between 10 and 20 and HUVECs in passages between 5 and 10 were used for the experiments.

### 2.2. Production of In Vitro Fracture Hematomas

In vitro fracture hematomas were prepared as previously described [20]. In brief, 60 µL of donor EDTA-blood was mixed with a solution of 1 × 10^6^ SCP-1 cells/mL in MEM-α, containing 10 mM CaCl_2_ (Sigma-Aldrich/Merck, Darmstadt, Germany), 5% FCS, and 1% penicillin/streptomycin (P/S) in non-coated 96-well plates. Donor EDTA-blood was collected from healthy male volunteers aged between 20 and 40 years. To induce coagulation, the plates were incubated for 1 h at 37 °C/5% CO_2_ in a humidified atmosphere. The hematomas were transferred to the respective experimental setup afterward.

### 2.3. Simulation of a Smoking Condition In Vitro

A combination of smoker’s blood and SCP-1 cells pre-stimulated with cigarette smoke extract (CSE) was used to mimic a smoking condition in an in vitro fracture hematoma, as described previously [20]. Blood was collected from moderately smoking male donors aged between 20 and 40 years. Before being added to in vitro fracture hematoma, SCP-1 cells were pre-stimulated with 5% CSE for 7 days, which is equivalent to a consumption of 10 cigarettes per day. CSE was prepared as described previously, using a gas wash bottle [38]. The selection of 5% CSE concentration was based on previous results from our group, which showed a visible effect on SCP-1 cells of this concentration level [39]. The medium change and CSE stimulation were repeated every 2 to 3 days.

### 2.4. Preparation of HUVECs Collagen I Sandwich Culture

To generate a 3D culture, HUVECs were seeded in 24-well plates in a collagen I (Col I) sandwich culture, following Ibidi’s application note 26 [40]. Col I was isolated from rat tail tendons, as described previously [41]. First, a Col I gel base with a Col I concentration of 3 mg/mL was produced. Therefore, 60% (v/v) Col I stock solution (5 g/L) was mixed with 6% (v/v) NaHCO_3_ solution (7.5% in ddH_2_0; Carl Roth, Karlsruhe, Deutschland), 7.3% (v/v) 10× M199 (Sigma-Aldrich/Merck, Darmstadt, Germany), and 26.7% (v/v) endothelial growth medium 2 microvascular (EGM-2 MV, PromocCell, Heidelberg, Germany). Gels were prepared in a sterile environment and solidified at 37 °C for 30 min. A total of 90,000 HUVEC cells per well were seeded in 450 µL culture medium. To ensure cell adherence only on the left side of the wells, the plates were tilted at a 25° angle for 2 h (37 °C, humidified atmosphere). Unattached cells were removed via quick washing with phosphate buffer saline (PBS), then 300 µL of a second Col I layer was applied at a Col I concentration of 1.5 mg/mL (30% (v/v) rat tail Col I, 6.7% (v/v) 10× M199, 2.6% (v/v) ddH_2_O, 3.7% (v/v) NaHCO_3_, and 3.3% (v/v) EGM2-MV). The solution was allowed to solidify for 30 min at 37 °C. A graphical representation of the experimental setup is further shown in Supplementary Figure S1.

### 2.5. Prescreening of Media Composition for the Co-Culture of In Vitro Fracture Hematomas and HUVECs

In vitro fracture hematomas were produced as described in Section 2.2. HUVECs were seeded in 96-well plates precoated with 0.1% gelatin. For media composition tests, in vitro fracture hematomas and HUVECs were separately cultured for 48 h at the following media ratios (in %) of EGM2-MV to MEM-α: 100:0, 75:25, 50:50, 25:75, 0:100; culturing was carried out in a hypoxia incubator chamber (STEMCELL Technologies, Vancouver, Canada) filled with a hypoxic gas mixture (1% O_2_, 5% CO_2_, 94% N_2_ (Westfalen, Münster, Germany)) in a humidified atmosphere at 37 °C. The viability and functionality tests were performed as described in the following sections. Due to the sensitivity of HUVECs, a 75:25 medium ratio was chosen for the co-culture. The results of the prescreening can be seen in Supplementary Figure S2.

### 2.6. Herbal Extracts and Prescreening of Stimulation Concentrations

Commercially available herbal extracts were obtained from Anklam Extrakte GmbH (Anklam, Germany). Based on information kindly provided by the supplier, both extracts were obtained via ethanolic extraction. The herbal extract of *P. ginseng* (GE, brand name: ginseng root powdered extract) was isolated from the plant roots and contains ≥10% of total ginsenosides, whereas the amount of main ginsenosides Rg1 and Rb1 is around 1% for both. The herbal extract of the berries from *Aristotelia chilensis*, also called maqui berries, (MBE, brand name: Delphinol^®^) contains ≥35% of total anthocyanins and ≥25% delphinidins. For cell culture, a stock solution of 1 mg/mL for GE and of 0.5 mg/mL for MBE was prepared in the respective culture medium and filtered sterile (0.22 µm) before use.

For screening of the appropriate herbal extract concentrations, HUVECs and in vitro fracture hematomas were separately cultured with extract concentrations of 0.1, 1, 10, 50, or 100 µg/mL each in 75:25 culture medium. The culture was carried out for 48 h in a hypoxia incubator chamber (STEMCELL Technologies, Vancouver, Canada) filled with a hypoxic gas mixture (1% O_2_, 5% CO_2_, 94% N_2_ (Westfalen, Münster, Germany)) in a humidified atmosphere at 37 °C. The viability and functionality tests were performed as described in the following sections.

### 2.7. Co-Culture of In Vitro Fracture Hematomas and HUVECs

To set up the co-cultures, three molds were punched on the right side of each Col I gel using a 5 mm biopsy puncher (Pfm Medical Mepro, Nonnweiler, Germany). Per mold, one in vitro fracture hematoma was placed. Cultures were cultivated either under control conditions with 75% EGM-2 MV: 25% MEM-α medium or with additional 10 µg/mL GE or 1 µg/mL MBE in a total volume of 1.5 mL medium per well. The culture was carried out in a hypoxia incubator chamber (STEMCELL Technologies, Vancouver, Canada) filled with a hypoxic gas mixture (1% O_2_, 5% CO_2_, 94% N_2_ (Westfalen, Münster, Germany)) for up to 48 h in a humidified atmosphere at 37 °C. Co-cultures were independently set up five times using blood from five non-smoking or smoking donors.

### 2.8. Mitochondrial Activity

Mitochondrial activity was assessed by resazurin conversion assay, always performed separately for in vitro fracture hematomas and HUVECs. For in vitro fracture hematomas, 100 µL of a 0.001% resazurin working solution (Sigma-Aldrich/Merck, Darmstadt, Germany) was added per hematoma and incubated at 37 °C for 90 min. Subsequently, 50 µL of the cell-free supernatant was measured. For HUVECs in 96-well plates or in a sandwich culture, respectively, 100 or 500 µL of resazurin working solution was added per well and incubated for 2 h at 37 °C. For both conditions, 100 µL of the cell-free supernatant was measured in 96-well plates at λEx = 544 nm/λEm = 590-10 nm, using an Omega plate reader (BMG Labtech, Ortenberg, Germany). Data are shown as relative fluorescence units (RFU).

### 2.9. Adenosine Triphosphate Assay

The adenosine triphosphate (ATP) content was determined using the CellTiter-Glo^®^ Luminescent Cell Viability Assay (Promega, Madison, WI, USA), following the manufacturer’s instructions. For analysis of the in vitro fracture hematomas, the incubation time was increased up to 30 min. The luminescence was determined using the Omega plate reader (BMG Labtech, Ortenberg, Germany). Data are shown as relative luminescence units (RLU).

### 2.10. Lactate Dehydrogenase Release

Lactate dehydrogenase (LDH) release in the culture medium was determined using CyQUANT™ LDH Cytotoxicity Assay (Thermo Fisher, Waltham, MA, USA), following the manufacturer’s instructions. Data are normalized to control conditions and shown as relative absorbance units (RAU).

### 2.11. Life Staining

Cells were stained with 0.1% Calcein-AM (Sigma-Aldrich, Darmstadt, Germany) for 20 min, at 37 °C, protected from light. The stain was removed, and cells were washed once with PBS. Images were acquired using the CELENA^®^ X High Content Imaging System (Logos Biosystems, Gyeonggi-do, Republic of Korea). Further analysis was performed using ImageJ software (Version 1.54d).

### 2.12. Alkaline Phosphatase Activity

To determine early osteogenic differentiation, alkaline phosphatase (ALP) activity of the in vitro fracture hematomas was determined by measuring the conversion of 4-Nitrophenyl phosphate (pNpp) to 4-Nitrophenol (pNp) [42]. The hematomas were incubated in 200 µL pNpp substrate solution (3.5 mM pNpp in 50 mM Glycine, 100 mM TRIS, 1 mM MgCl_2_, pH 10.5 (all Carl Roth, Karlsruhe, Germany)) for 60 min at 37 °C. Afterward, the absorbance at λ = 405 nm was measured in the cell-free supernatant using the Omega plate reader. ALP activity was normalized to ATP content.

### 2.13. Total Protein Staining

Total protein content was determined via sulforhodamine B (SRB) staining. The ethanol-fixed cells were washed once with tap water before being covered with the SRB staining solution (0.4% (w/v) SRB in 1% acetic acid solution; Sigma-Aldrich/Merck, Darmstadt, Germany) and incubated at room temperature for 30 min, protected from light. Subsequently, the unbound dye was removed by washing with a 1% acetic acid solution. For quantification, the bound stain was dissolved in 10 mM TRIS unbuffered (Sigma-Aldrich/Merck, Darmstadt, Germany) and measured with the Omega plate reader. Measurement values (λ = 565 nm) were background corrected by subtracting values obtained at λ = 690 nm.

### 2.14. Gene Expression Analysis

RNA from the in vitro fracture hematomas was isolated using chloroform/phenol extraction [20]. RNA from HUVECs in Col I gels was isolated from the pooled samples using chloroform/phenol extraction followed by purification of the aqueous phase with the All-in-One Prep Kit (Biotrend, Köln, Germany), as per the manufacturer’s instructions. The integrity of the isolated RNA was verified by agarose gel electrophoresis. cDNA was synthesized using the First-strand cDNA Synthesis kit (Thermo Fisher, Waltham, MA, USA). The reverse transcription polymerase chain reactions (RT-PCRs) were carried out in a total volume of 15 µL, consisting of 7.5 µL Red HS Master Mix (Biozym, Hessisch Oldendorf, Germany), 7.5 µL primer reverse and forward, and, respectively, either 1 or 2 µL of cDNA template and filled up with RNase/DNase-free water. The PCRs were run appropriately for the used Master mix in an Applied Biosystems™ Veriti™ Thermal Cycler (Thermo Fisher, Waltham, MA, USA). Primer details and cycling conditions are listed in Table 1. Amplification was visualized using agarose gel electrophoresis, whereas each PCR was visualized twice to minimize loading differences. Gene expression analysis of the in vitro fracture hematomas was performed for each donor separately (N = 5), and that of HUVECs was carried out with pooled samples and therefore was repeated three times (N = 3). The band intensities were determined using ImageJ software. All PCRs were normalized to housekeeping gene Eukaryotic translation elongation factor 1 alpha 1 (EF1α).

### 2.15. Enzyme-Linked Immunosorbent Assay

Cytokine secretion for TNF-α, IL-6, and CCL2 was determined using enzyme-linked immune sorbent assay kits (ELISAs) from Peprotech (Hamburg, Germany), following the manufacturer’s instructions (Article numbers: TNF-α: 900-K25; IL-6: 900-K16; CCL2: 900-K31).

### 2.16. HUVEC Tube Formation Assay

First, 6.5 × 10^4^ HUVECs were seeded in 6 µL GeltrexTM (Thermo Scientific, Waltham, MA, USA) pre-coated wells of a 48-well plate in a thin-layer angiogenesis assay [20,43]. The respective culture supernatants for stimulation were diluted 1:1 in EBM-2 (PromocCell, Heidelberg, Germany) to a combined volume of 200 µL. The cells were incubated at 37 °C, 5% CO_2_ for 18 h in a humidified atmosphere, and stained with 0.1% Calcein-AM for 10 min at 37 °C. Tube formation was assessed by means of the pooled culture supernatants in three biological (N = 3) and technical replicates (n = 3). Images were acquired using the CELENA^®^ X High Content Imaging System (Logos Biosystems, Gyeonggi-do, Republic of Korea) and analyzed with the ImageJ Angiogenesis analyzer plugin [44]. For simplification, all data were normalized to non-smokers.

### 2.17. Statistics

Statistical analyses were made using GraphPad Prism 8 (San Diego, CA, USA). Non-parametric Mann–Whitney tests or Kruskal–Wallis tests, followed by Dunns’ multiple comparisons, were used to compare two or more experimental groups, respectively. The smoker and non-smoker conditions were initially compared to each other. Later, the unstimulated smoker condition was additionally compared to the conditions stimulated with GE and MBE. The number of biological replicates (N) and technical replicates (n) is given in the figure legend for each performed experiment. Levels of significance were defined as * *p* < 0.05, ** *p* < 0.01, *** *p* < 0.001. Data are usually shown as Tuckey blots, where small black dots indicate the outliers. Gene expression data are shown as bar graphs, displaying the mean ± standard error of the mean (SEM).

## 3. Results

### 3.1. GE and MBE Were Not Toxic for the In Vitro Fracture Hematomas and HUVECs in Concentrations up to 100 µg/mL

**Figure 1:**
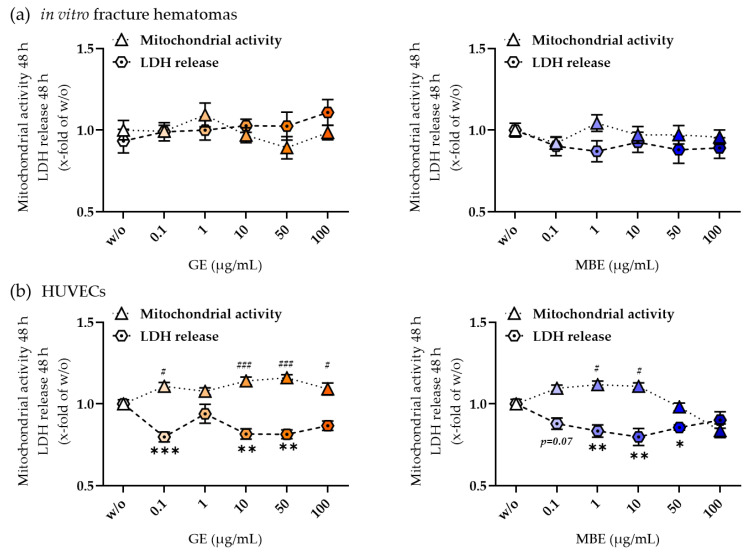
Toxicity test of herbal extracts. With mitochondrial activity and LDH release of in vitro fracture hematomas (**a**) and HUVECs (**b**) stimulated with 0, 0.1, 1, 10, 50, or 100 µg/mL MBE or GE for 48 h. Mitochondrial activity is always shown as triangles, whereas LDH release as hexagons. Data were normalized to w/o. Statistics were made comparing to w/o only. Experiments were performed in N = 4, n = 3. Data are shown as mean ± SEM. Levels of significance were defined as * ^or #^ *p* < 0.05, ** *p* < 0.01, *** ^or ###^ *p* < 0.001, and are shown as asterixis (*) for LDH release and as hashes (#) for mitochondrial activity.

The toxicity of herbal extracts was assessed via measurement of mitochondrial activity and LDH release in both co-culture components, the in vitro fracture hematomas, and HUVECs, separately. As displayed in Figure 1a, both extracts did not influence the viability of hematomas in all tested concentrations. HUVECs showed a decrease in LDH release and an increase in mitochondrial activity in the culture concentrations of 0.1, 1, 10, and 50 µg/mL, with an exception for 1 µg/mL GE (Figure 1b). As can be seen in Supplementary Figure S3, a stimulation with MBE in concentrations of 1 and 10 µg/mL also increased the HUVECs’ total protein content, whereas the protein content of the GE-stimulated HUVECs did not change. Therefore, both MBE and GE showed a positive influence on the proliferation of HUVECs at lower concentrations of the tested range. Based on the literature and our previous studies, concentrations of 10 µg/mL GE and 1 µg/mL MBE were chosen for further analysis [23,24].

### 3.2. GE and MBE Could Not Decrease Stress Levels in the Smoker In Vitro Fracture Hematomas

**Figure 2:**
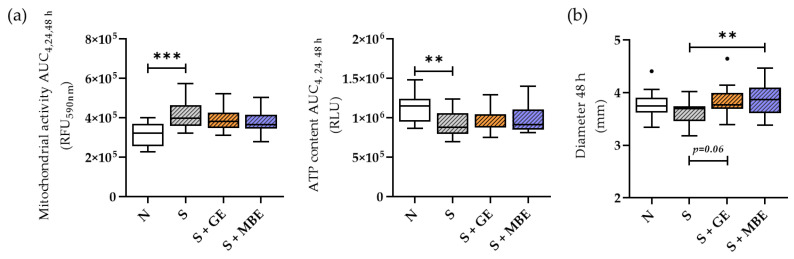
Viability of the non-smoker (N) and smoker (S) in vitro fracture hematomas in the co-culture with HUVECs stimulated with ginseng extract (+GE) or maqui berry extract (+MBE). With (**a**) mitochondrial activity and ATP content, shown as area under the curve (AUC) for measurement time points 4, 24, and 48 h, and (**b**) diameter of the in vitro fracture hematomas after 48 h of incubation. All data are shown as Tuckey blots and collected in N = 5, n = 3. Outliers are represented by small black dots. Levels of significance were defined as ** *p* < 0.01, *** *p* < 0.001.

The non-smoker and the smoker co-cultures were assembled. The smoker co-cultures were further stimulated with either GE or MBE. The viability of the in vitro fracture hematomas of all conditions was analyzed by means of mitochondrial activity, ATP content, and size (diameter). The results are displayed in Figure 2. The smoker in vitro fracture hematomas showed higher mitochondrial activity and lower ATP content than the non-smoker fracture hematomas. Stimulation with MBE and GE influenced neither the ATP content nor the mitochondrial activity of the smoker hematomas. The increase in mitochondrial activity, accompanied by the reduction in the ATP content, revealed a potential increase in the oxidative stress in our smoker hematomas (Figure 2a). Despite a high content of antioxidative phytochemicals, neither extract was able to counteract a potential higher oxidative stress. However, GE and MBE increased fracture hematoma diameters in ranges similar to the non-smoker conditions (Figure 2b).

### 3.3. Higher Inflammatory Status of Smoker Cultures Could Not Be Reduced by GE or MBE

**Figure 3:**
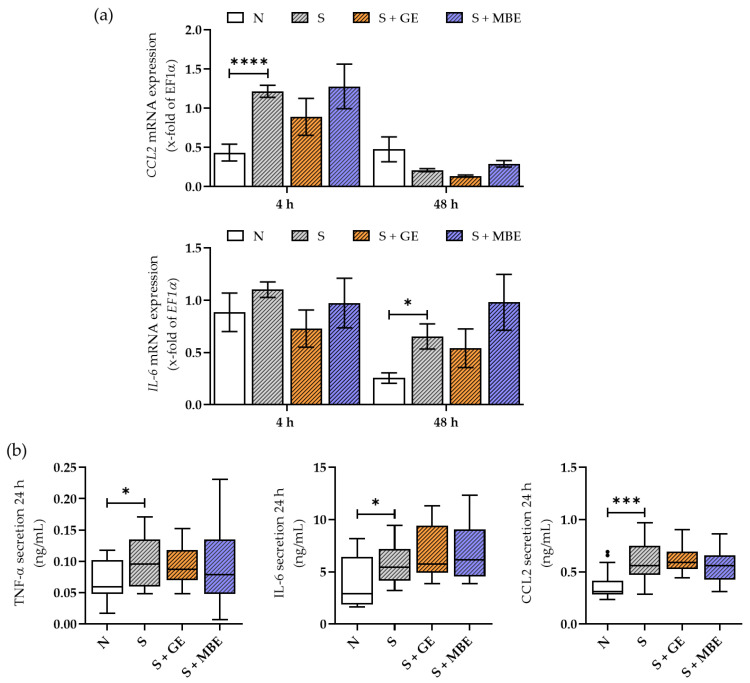
The inflammatory status of the non-smoker (N) and smoker (S) co-cultures additionally stimulated with ginseng extract (+ GE) or maqui berry extract (+ MBE). With (**a**) mRNA expression of pro-inflammatory cytokines *CCL2* and *IL-6* after 4 and 48 h in the in vitro fracture hematomas. N = 5, n = 2. Data are shown as mean ± SEM. Statistics were made for each time point separately. (**b**) Secretion of *TNF-α*, *IL-6*, and *CCL2* in co-culture supernatants after 24 h of incubation. N = 5, n = 3. Data are shown as Tuckey blots. Outliers are represented as small black dots. Levels of significance were defined as * *p* < 0.05, *** *p* < 0.001, **** *p* < 0.0001.

Being one of the main drivers of early fracture healing, the inflammatory status was previously shown to be increased in smokers and was analyzed in the in vitro fracture hematomas as well. The hematomas of smokers showed higher expression of *CCL2* (after 4 h) and *IL-6* (after 48 h), as can be seen in Figure 3a. Although the stimulation with GE showed a trend in reducing cytokine expression of *IL-6* and *CCL2*, the results were not significant. Stimulation with MBE did not alter the mRNA levels of either of the tested cytokines.

The inflammatory status was further analyzed in co-culture supernatants, which considers the secretion by the in vitro fracture hematomas and the HUVECs (Figure 3b). In all co-culture supernatants, a pattern similar to the previously shown gene expression analysis of the in vitro fracture hematomas was detected. The smoker co-cultures showed higher levels of secreted pro-inflammatory cytokines after 24 h, which were not influenced by stimulation with MBE and GE. It has to be noted that the overall secretion of cytokines in cell culture supernatants partially leveled out after 48 h of incubation, which can be seen in Supplementary Figure S4. In summary, both extracts could not decrease the higher inflammatory status of cultures mimicking the in vitro fracture repair of the smokers.

### 3.4. Reduced Osteogenic Potential of the Smoker In Vitro Fracture Hematomas Could Not Be Promoted by Stimulation with GE or MBE

**Figure 4:**
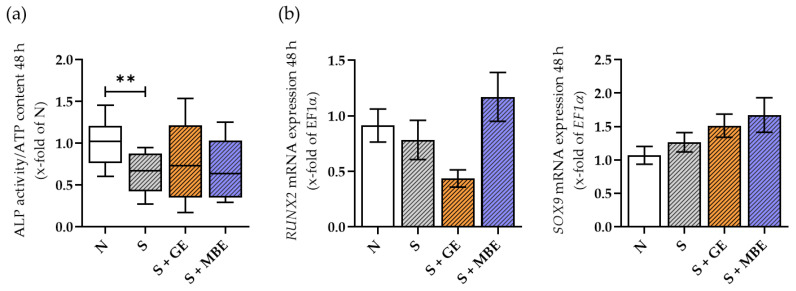
Osteogenic and chondrogenic differentiation potential of the non-smoker in vitro fracture hematomas (N) and of the smoker in vitro fracture hematomas (S) additionally stimulated with ginseng extract (+GE) or maqui berry extract (+MBE) after 48 h of incubation. With (**a**) ALP activity normalized to the non-smoker conditions. N = 5, n = 3. Data are shown as Tuckey blots. (**b**) Gene expression of *RUNX2* and *SOX9*. N = 5, n = 2. Data are shown as mean ± SEM. Levels of significance were defined as ** *p* < 0.01.

The differentiation of MSCs is crucial for the onset of healing. Within the co-culture model, the early osteogenic and chondrogenic differentiation events of the in vitro fracture hematomas were analyzed by means of ALP activity as well as gene expression of osteogenic transcription factor *Runt-related transcription factor 2 (RUNX2)* and chondrogenic transcription factor *SRY-Box Transcription Factor 9 (SOX9)*. Results are shown in Figure 4. ALP activity was reduced in the smoker in vitro fracture hematomas, whereas *RUNX2* and *SOX9* gene expression did not differ between the smoker and non-smoker cultures. Treatment with MBE could not rescue the ALP activity of the smoker in vitro facture hematomas and showed a slight trend in inducing *RUNX2* and *SOX9* expression. Nevertheless, the results were not significant. GE did not affect ALP activity and *SOX9* expression in the smoker culture but showed a trend in reducing *RUNX2* expression, though also not significantly. In summary, both extracts did not induce osteogenic or chondrogenic potential in the smoker in vitro fracture hematomas.

### 3.5. HUVECs’ Increased Stress in the Smoker Co-Cultures Could Not Be Reversed by GE or MBE

**Figure 5:**
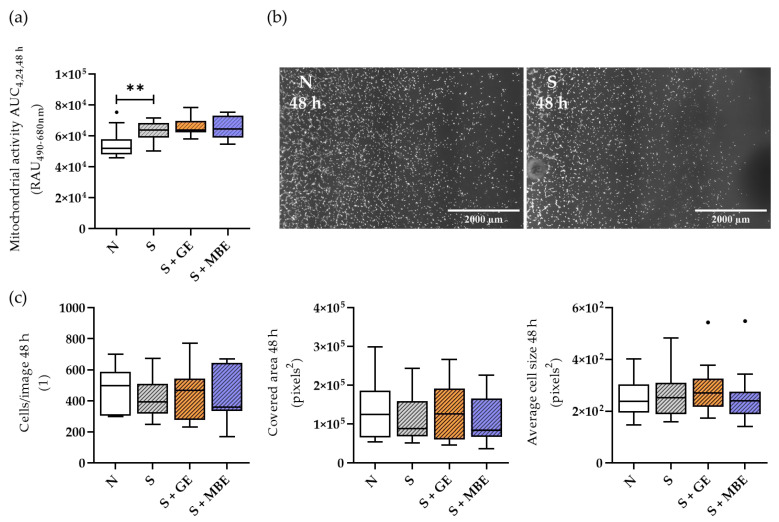
HUVECs’ viability in the non-smoker (N) and smoker (S) co-cultures, additionally stimulated with ginseng extract (+GE) or maqui berry extract (+MBE). With (**a**) mitochondrial activity of HUVECs shown as AUC for measurement time points 4, 24, and 48 h. (**b**) Representative life-staining images after 48 h of incubation for the smoker (S) and non-smoker (N) conditions. (**c**) Particle analysis of life-staining images after 48 h of incubation with cells/image, covered area, and average cell size. N = 5, n = 3. Per well, particles at three identical positions were analyzed, and results were averaged (n). Data are shown as Tuckey blots. Outliers are represented as small black dots. Levels of significance were defined as ** *p* < 0.01.

Since only small influences of the herbal extracts could be observed in the early fracture repair of smokers in regard to inflammation, as well as osteogenic and chondrogenic differentiation potential, we decided to take a closer look at the endothelial component of the culture system—the HUVECs—initially testing the viability. The results are shown in Figure 5. HUVECs in co-culture with the smoker in vitro fracture hematomas showed higher mitochondrial activity (Figure 5a). To differentiate whether higher mitochondrial activity was related to a higher number of cells or rather to higher cellular stress, the evaluation of life-staining images of HUVECs was carried out. The analysis of the number of cells, the covered area, and the average cell size did not reveal any differences between the smoker and non-smoker conditions (Figure 5b,c). This led us to the assumption that the higher mitochondrial activity of HUVECs in the smoker cultures may reflect higher cellular stress rather than an increase in cell proliferation. Stimulation with both herbal extracts neither influenced the mitochondrial activity of the smoker HUVECs nor the number of cells, the covered area, or the average cell size. In summary, none of the herbal extracts were able to reduce the smoking-induced oxidative stress in HUVECs.

### 3.6. Abolished Expression of Pro-Inflammatory Cytokines and CD31 in HUVECs in the Smoker Cultures Could Be Partially Reversed by GE but Not by MBE

During the inflammation, the endothelium perpetuates it by secreting pro-inflammatory cytokines, thereby recruiting additional immune cells and promoting the progression of healing [45].

**Figure 6:**
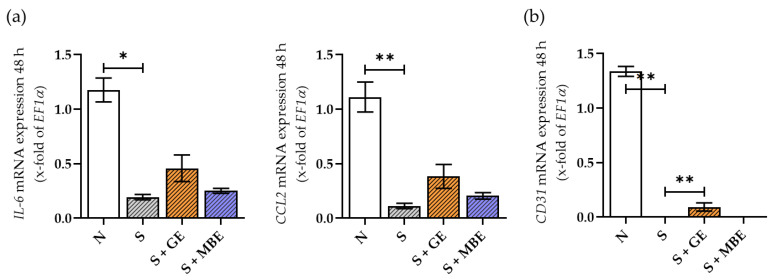
Gene expression analysis of pro-inflammatory cytokines CCL2, IL-6 (**a**), and a surface marker CD31 (**b**) in HUVECs from the non-smoker (N) and smoker (S) co-cultures, additionally stimulated with ginseng extract (+GE) or maqui berry extract (+MBE) after 48 h of incubation. N = 3, n = 2. Data are shown as mean ± SEM. Statistics were made for each time point separately. Levels of significance were defined as * *p* < 0.05, ** *p* < 0.01.

In HUVECs, the analysis of gene expression of pro-inflammatory cytokines *IL-6* and *CCL2* revealed a contrasting picture to the results obtained in the in vitro fracture hematomas. Overall, the expression and secretion of pro-inflammatory cytokines were reduced in the smoker conditions. The results are displayed in Figure 6a. Although GE showed a clear tendency to increase the expression of pro-inflammatory cytokines, the results were not significant.

The growing blood vessels in bone and fracture environments have been identified to have high expressions of the cluster of differentiation 31 (*CD31*) and *Endomucin* [46,47]. As a representative of the intact growing vessels, we analyzed the gene expression of *CD31*. The smoking conditions completely nullified the gene expression of endothelial marker *CD31* in HUVECs. Incubation with GE significantly increased the expression of *CD31* in the smoker HUVECs within the first 48 h, while MBE had no effect (Figure 6b).

Overall, HUVECs in the smoker co-cultures seem to be impaired in their function, since they were not able to adapt to the inflammatory environment via cytokine expression and further showed a complete abolishment of *CD31* expression. Nevertheless, stimulation with GE showed a clear tendency to be able to reverse the smoking-induced damage.

### 3.7. GE Increased the Smoker HUVECs’ Angiogenic Potential along the Angpt/Tie2 Axis while Suppressing the VEGF Signaling

**Figure 7:**
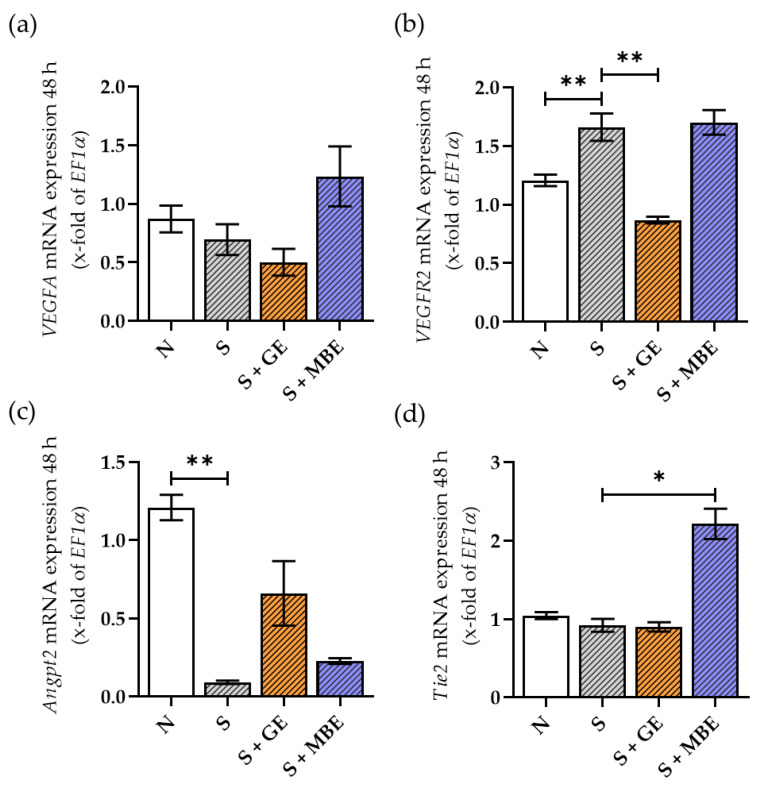
Gene expression of *VEGFA* (**a**), *VEGFR2* (**b**)*, Angpt2* (**c**), and *Tie2* (**d**) in HUVECs after 48 h of incubation in co-culture with non-smoker in vitro fracture hematomas (N) and smoker in vitro fracture hematomas (S), additionally stimulated with ginseng extract (+GE) or maqui berry extract (+MBE). Data are shown as mean ± SEM. Levels of significance were defined as * *p* < 0.05, ** *p* < 0.01.

In both healthy and pathological angiogenesis, VEGF signaling is the most often studied pathway. Recently, we showed that Angpt/Tie2 signaling is dysregulated in the early fracture healing of smokers [20]. Therefore, in the present study, we analyzed the effects of both herbal extracts on VEGF and Angpt/Tie2 signaling. Both pathways were analyzed in HUVECs through the gene expression of ligands *VEGFA* and *Angpt1/2* and their respective receptors, *vascular endothelial growth factor receptor* (*VEGFR) 2* and *Tie2*. The results are displayed in Figure 7. No differences in VEGF expression could be observed between the smoker and non-smoker HUVECs. MBE showed a tendency to induce *VEGFA* gene expression in the smoker HUVECs, though the results were not significant (Figure 7a). *VEGFR2* expression was increased in the smoker HUVECs. While MBE had no effect, GE significantly downregulated the receptor’s expression (Figure 7b). Regarding Angpt signaling, the expression of *Angpt1* could not be detected at all in HUVECs. Nevertheless, the smoker HUVECs showed a harsh reduction in *Angpt2* expression, which was partially restored by GE but not by MBE (Figure 7c). In contrast, MBE but not GE increased the smoker HUVECs’ *Tie2* expression (Figure 7d). In summary, MBE showed a diverse regulation of angiogenesis in HUVECs. At the same time, GE induced angiogenic potential in the smoker in vitro fracture hematomas along the *Angpt/Tie2* axis while suppressing the VEGF pathway.

### 3.8. GE Increased the Smoker In Vitro Fracture Hematomas Angiogenic Potential along the Angpt/Tie2 Axis Meanwhile Suppressing the VEGF Signaling

**Figure 8:**
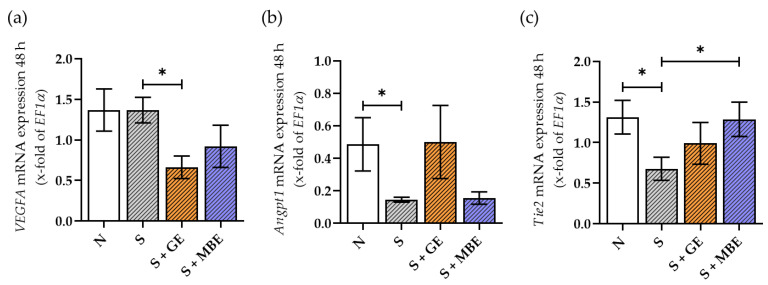
Angiogenic potential of the non-smoker in vitro fracture hematomas (N) and smoker in vitro fracture hematomas s (S), additionally stimulated with ginseng extract (+GE) or maqui berry extract (+MBE) after 48 h of incubation, determined by gene expression analysis of *VEGFA* (**a**), *Angpt1* (**b**), and *Tie2* (**c**). N = 5, n = 2. Data are shown as mean ± SEM. Levels of significance were defined as * *p* < 0.05.

Furthermore, the angiogenic potential of the in vitro fracture hematomas was analyzed in regard to the VEGF and Angpt signaling pathways through gene expression analysis. The results are displayed in Figure 8. The smoker and non-smoker in vitro fracture hematomas showed similar levels of *VEGFA* expression, but stimulation with the herbal extracts reduced *VEGFA* expression in the smoker hematomas (Figure 8a). *VEGFR2* as well as *Angpt2* expression could not be detected at all in the in vitro fracture hematomas. Regarding the Angpt/Tie2 axis, smoking conditions reduced the expression of *Angpt1* in the hematomas, which could be restored to the non-smoker levels via GE stimulation (Figure 8b). While smoking conditions and GE showed no influence on *Tie2* expression in the hematomas, MBE could further increase it (Figure 8c).

In summary, MBE showed no clear trend in supporting angiogenesis in the in vitro fracture hematomas, whereas GE induced the angiogenic potential along the Angpt/Tie2 axis while suppressing the VEGF pathway.

### 3.9. MBE Further Impaired the Already Diminished Tube Formation Potential of the Smoker Cultures

**Figure 9:**
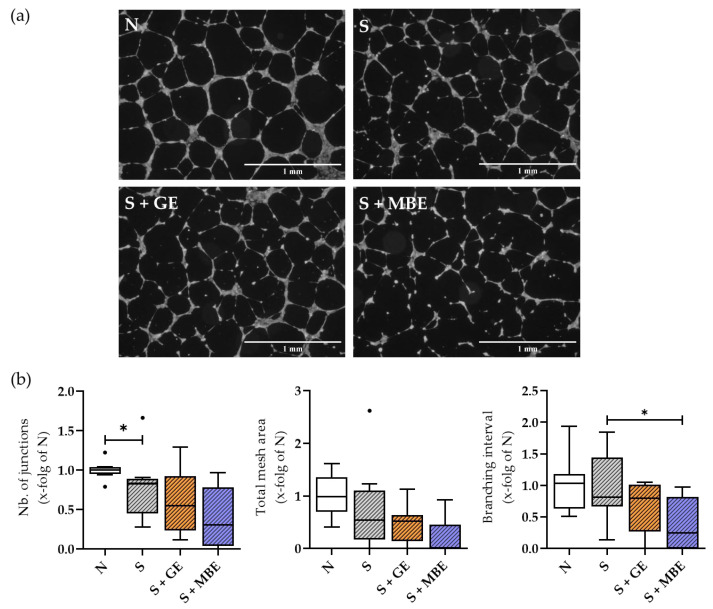
The ability of culture supernatants of the non-smoker (N) and smoker (S) cultures, additionally stimulated with ginseng extract (+GE) or maqui berry extract (+MBE) after 48 h of incubation to induce HUVEC tube formation. With (**a**) representative microscopic images recorded in 4× magnification. Scale bar represents 1 mm. (**b**) Evaluation of images with the number of junctions, total mesh area, and branching interval. N = 3 (pooled), n = 3. Per well (n), tube formation at three identical positions was analyzed and results were averaged. Data are shown as Tuckey blots. Outliers are represented as small black dots. Levels of significance were defined as * *p* < 0.05.

Lastly, the effects of culture supernatants on HUVEC tube formation were assessed to identify their influence on the surrounding vasculature. The results are shown in Figure 9. The smoker culture supernatants resulted in an improper mesh formation, with fewer junctions formed. Whereas the stimulation with GE did not alter the tube formation ability of HUVECs, the stimulation with MBE additionally impaired the formation of a proper network, leading to reduced branching intervals, as well as to a reduction in the mesh area and in the number of junctions formed (Figure 9b). In summary, GE did not affect tube formation, whereas MBE further impaired the tube-forming ability of the smoker cultures.

## 4. Discussion

The herbal extracts of maqui berries and ginseng roots have been described in the literature for their beneficial effects on bone welfare. Ginseng extracts were further shown to improve fracture repair in several studies with rodents, whereas research on the possible role of MBE in fracture repair is relatively rare [48,49]. The consumption of cigarettes is one of the major risk factors for developing a fracture healing disorder, affecting several aspects of fracture repair, including inflammation, osteogenesis, and angiogenesis. In a previous study, we could demonstrate that the deleterious effects of CSE could be reversed by treatment with GE and MBE in a co-culture model of osteoblastic and osteoclastic cells [24]. Within this study, we wanted to prove that these two herbal extracts could also support the early fracture repair of smokers, previously shown to be strongly impaired in vitro [20]. For analysis, a 3D co-culture model, consisting of the smoker in vitro fracture hematomas and HUVECs, was used to analyze the fracture hematoma and its influence on early angiogenic events and vice versa.

Early fracture healing in smokers was accurately modeled, and showed very similar results as previously published in in vitro and in vivo studies [19,20,21]. The smoker co-cultures showed an overall higher inflammatory status, a reduction in osteogenic differentiation potential, and impaired angiogenesis. In a smoking mice model, Hao et al. reported an overall increase in inflammatory markers, including CCL2 and IL-6 up to 24 h after fracture, as well as a decreased recruitment of stem cells accompanied by reduced callus formation and impaired angiogenesis [21].

Commonly described to prevent cardiovascular diseases, both extracts have been associated with a reduction in platelet activation and thrombus formation [50,51]. In more detail, for instance, ginsenosides Rg2 and Rg3 inhibited clotting factor coagulation [52], or delphinidin-3-glucoside (Dp-3-g) altered platelet cell surface expression of markers p-selectin, CD63, and CD40L, thereby blocking fibrin binding [53]. An altered fibrin clot structure is therefore most likely upon stimulation with GE and MBE. The observed larger diameters of the in vitro fracture hematomas highlight a diverging fibrin clot formation. Still, a deeper analysis of the fibrin network is necessary, to prove the hypothesis and to identify its influence on healing outcomes.

Smoking is commonly associated with cellular stress caused by intracellular reactive oxygen species (ROS) formation [54]. Herbal extracts of *P. ginseng,* and especially of maqui berry, are rich in phytochemicals attributed to antioxidative properties [28,32]. Goszcz et al. reported that physiological concentrations of delphinidin (10 µM), the most prominent anthocyanin of maqui berries, were able to counteract externally applied oxidative stress and increased intracellular glutathione contents in HUVECs [55]. Furthermore, similar dosages of the same maqui berry extract used in our experiment have also been shown to prevent cigarette-smoke-induced oxidative stress in human osteoblasts via the induction of phosphorylated nuclear factor erythroid 2-related factor 2 (pNRF2) signaling [23]. MBE and GE in the presented in vitro model system were not able to reduce the possible higher oxidative stress of either the smokers in vitro fracture hematomas of HUVECs. It must be noted that in the co-culture system, possible oxidative stress was only detected indirectly by the increased mitochondrial activity, which was refuted by other viability measurements. Thus, further analysis is necessary to prove and confirm oxidative stress levels in the cultures. As oxidative stress and inflammation are tightly coupled, antioxidative and anti-inflammatory properties are often linked. It is therefore not surprising that MBE and GE have both been widely shown to reduce and inhibit cytokine production of, for instance, TNF-α, IL-1β, IL-6, and interferon-gamma (IFN-γ) [56,57]. Moreover, MBE, as well as Rg3, were reported to actively contribute to the resolution of inflammation by reducing the pro-inflammatory responses of macrophages and also by promoting their polarization from M1 to M2 [58,59]. Concerning smoking, the extracts were mostly described for their positive effects on airway epithelial inflammation. For instance, MBE normalized hydrogen peroxide (H_2_O_2_) and IL-6 concentrations in smokers’ breath condensates [60], and GE given 1 h post 7 days of CSE exposure inhibited both recruitment of immune cells and release of pro-inflammatory cytokines TNF-α, IL-6, and CCL2 in the airway epithelium [61]. It was surprising that neither GE nor MBE reduced the inflammation in the smoker in vitro fracture hematomas or HUVEC cells in our in vitro model of early fracture repair. However, the results are in line with the possible increased oxidative stress, which could also not be prevented by the extracts.

Ginseng extracts and ginsenosides are often described in the literature to induce osteogenic differentiation both in vitro and in vivo. For instance, 0.1, 1, and 10 µg/mL ginsenoside Rb1 or 50 µM of ginsenoside Re were shown to increase RUNX2 and ALP protein levels after 7 days in osteoprogenitor cells [49,62]. It was also demonstrated that the same MBE used in this study induced the gene expression of osteogenic factors, including *RUNX2*, and ALP activity in osteoprogenitor cells after four days of stimulation, although the effect was concentration-dependent and only the highest concentration of 25 µg/mL was found to be effective [34]. Reported effects of the extracts on chondrogenesis are rare, though anthocyanin-rich pomegranate fruit extract has been shown to act inductively on *SOX9* expression [63]. With regard to the inflammatory milieu in osteoporosis, both extracts have been reported to further support bone homeostasis by inhibiting osteoclastogenesis, thereby promoting bone formation [24,33,64]. In our previous study, GE (10 µg/mL) and MBE (1.5 µg/mL) proved to be able to reverse the CSE-induced reduction in *RUNX2* and *ALP* expression after 7 days of incubation [24]. In contrast to that, in our study, both herbal extracts were not able to reverse osteogenic or chondrogenic potentials in the smoker in vitro fracture hematomas. Generally, it has to be remarked that for the analysis of osteogenic differentiation in fracture repair, longer incubation times would be preferable, since an increase in osteogenic differentiation potential is usually observed between 48 and 72 h post-fracture [65,66].

Both extracts were reported to have effects on angiogenesis, however, mainly without focusing on repair processes. As a prospective cancer treatment, extracts and phytochemicals of maqui berries were commonly described as anti-angiogenic, and 10 µM of delphinidin were shown to hamper HUVEC tube formation via the inhibition of VEGFR2 downstream signaling [35,36,67]. In the presented model, MBE also showed a negative effect on angiogenesis, as it further reduced HUVEC tube formation, which was already impaired by smoking conditions. Apart from this, the extract had rather little effect, except for its ability to increase *Tie2* gene expression. Further analyses are necessary to understand the interference as a whole, also supported by the scarcity of corresponding studies, especially regarding the regulation of *Tie2* expression and its effects [20]. In contrast to that, ginsenosides and ginseng extracts are usually described to be pro-angiogenic. Saponin extracts of *P. ginseng* (50–100 µg/mL) were shown to increase VEGFA in rat bone marrow MSCs, HUVEC proliferation, and tube formation, as well as vascularization in a zebrafish model [27,68]. In our study, stimulation with GE could not promote HUVECs tube formation in the smoker conditions but increased expression of growth factors *Angpt1* and *2* to non-smoker levels. Supportive effects of ginseng on the Angpt/Tie2 system were reported previously. Ginsenoside Notoginseng R1 (100 µM) was shown to induce angiogenesis via endothelial cell migration and vessel sprouting by means of increased autocrine Angpt2 production and consequent activation of the Tie-2 signaling [69]. GE could therefore support impaired angiogenesis in smokers by promoting the Angpt/Tie2 axis.

The type of vasculature, formed within fracture environments, is usually characterized by the high expression of CD31 and Endomucin, also classified as type H vessels, which are also important in coupling angiogenesis and osteogenesis [46,70]. Under smoking conditions, CD31 expression in HUVECs was completely abolished. Whereas MBE did not support the expression, GE potentially induced CD31 in the smoker HUVECs. The effect of MBE on vessels in the fracture environments is not known from the current literature, but anthocyanins have been reported to suppress CD31 expression in tumor environments and platelets [51,71]. On the contrary, type H vessel formation in rodents as well as the expression of CD31 and Endomucin in endothelial cells have been shown to be supported by different ginseng root extracts and ginsenosides, including *P. quinquefolium* saponin (PQS, from American ginseng), saponin Ophiogonin D, or ginsenoside Rg1 [47,72,73].

Surprisingly, both herbal extracts had, overall, rather small impacts on the early fracture repair of smokers in vitro, as they were not able to reduce the prolonged inflammatory state or to increase the osteogenic or chondrogenic differentiation potentials. Still, GE could partially promote early angiogenic events, including the Angpt/Tie2 system dysregulated in early fracture repair of smokers, as well as CD31 expression of the smoker HUVECs. In conclusion, MBE and GE did not strongly support early fracture repair of smokers in the used model system. Additionally, comparative literature, especially regarding MBE and its use in inflammatory environments, is rare.

The use of herbal extracts in research is also subject to some restrictions and limitations. In general, it is quite challenging to compare studies on herbal extracts due to the general lack of detailed information on the exact composition of extracts and, therefore, to their possible differences. For instance, extracts from *P. notoginseng* have three times higher saponin contents than extracts obtained from *P. ginseng*, and it is not known if differences attributed to the two different extracts are caused by the generally higher saponin content or by other factors [30]. Therefore, studies focusing on only one specific ginsenoside or anthocyanin are more suitable for comparisons. At the same time, the complexity of mixtures is also an advantage, since the biological activity of extracts may also be a result of the combination of several compounds acting synergistically [74]. In this work, standardized commercially available extracts were used, which can partially overcome this diversity. Still, the exact chemical composition of the used extracts is also so far unknown. Even though comparison is difficult, it is, when carefully conducted, valid and can give insights into possible mechanisms.

Since the observed effects were rather small, it could be considered if higher doses would have had more significant effects. Compared to other in vitro studies, the dosages used in the presented experiments are indeed rather small. Our choice of dosage was based on our previous studies, where the same dosages could counteract smoking-induced damage [23,24]. Comparing in vitro and in vivo data on dosages is more complex, as extracts are mostly applied orally. The bioavailability of extracts is reported to be around 10% for ginsenosides but only 1% for anthocyanins [75,76]. The oral administration of 9 g GE with a total of 45.81 mg ginsenoside Rb1 led to a peak concentration of approximately 4 ng/mL in human plasma after 10 h. The main metabolite compound K reached a maximum concentration of around 8.5 ng/mL after 10 to 15 h [77]. Plasma concentrations of MBE have been shown to range between 1 and 1000 nM and were reached between 1 and 4 h post intake [78,79]. For sure, in further experiments, the comparison of different concentrations and application strategies of herbal extracts would also be interesting. For example, pre-stimulation could be a useful approach to mimic preventive ingestion of the herbal extracts before fractures can even occur. Nevertheless, the applied concentrations range at physiological levels. Furthermore, high dosages of antioxidants are not necessarily preferable. Several antioxidants are associated with pro-oxidant and pro-inflammatory properties, which are favored by high dosages, the presence of transition metal ions, or generally influenced by the antioxidants’ redox potential [80]. For instance, supraphysiological concentrations of delphinidin (100 µM) were shown to be toxic for HUVEC cells, due to the development of pro-oxidative components [55].

Lastly, one of the major limitations of the used in vitro system is its limited maximal lifetime of 48 h. The analysis of longer incubation times would be preferable to evaluate long-term outcomes.

In comparison to previous studies, herbal extracts may have greater benefits when administered for longer periods and/or in later stages of the healing process. Though, in summary, both herbal extracts also did not negatively influence the early fracture repair processes of smokers in vitro.

## 5. Conclusions

Within the co-culture model, smoking-impaired early fracture healing processes are similar to the in vivo observations in humans and mice. Smokers showed an increased inflammatory status, as well as impaired osteogenesis and angiogenesis. Herbal extracts of *P. ginseng* and maqui berry in the tested dosages had a rather small impact on the smoker early fracture healing in vitro, as they were not able to reduce inflammation or increase osteogenesis and, for MBE only, angiogenesis. However, ginseng extract showed a tendency to promote angiogenesis along the Angpt/Tie2 axis, which has been shown in previous experiments to be a candidate for the dysregulation of angiogenesis during early fracture repair in smokers in vitro. Although no outstanding benefits were reported, the treatment with GE also had no drawbacks, such as a complete suppression of the initial inflammatory responses. Treatment with MBE impaired angiogenesis. Based on the current literature, herbal extracts may be more likely to promote later phases of fracture healing, including bone remodeling, by reducing osteoclast formation or inducing angiogenesis at later stages.

## Figures and Tables

**Table 1:** RT-PCR primer details and cycling conditions. Conditions are shown for in vitro fracture hematomas. Diverging conditions for HUVECs are shown in brackets.

Target and Accession Number	Primer Sequence (5′ to 3′) ^1^	Fragment Length	T_An_^2^	n_Cyc_^3^	m_cDNA_^4^
*Anpgt1* NM_001146.5	F CGATGGCAACTGTCGTGAGA R CGATGGCAACTGTCGTGAGA	232 bp	60 °C	35	20 ng
*Angpt2* NM_001147.3	F CTTGGAACACTCCCTCTCGAC R GCTTGTCTTCCATAGCTAGCAC	125 bp	60 °C	32 (40)	20 ng (10 ng)
*CCL2* NM_002982.3	F CCTTCATTCCCCAAGGGCTC R CCTTCATTCCCCAAGGGCTC	236 bp	60 °C	35	20 ng
*CD31* NM_000442.4	F GATAGCCCCGGTGGATGA R GTTCCATCAAGGGAGCCTTC	726 bp	60 °C	28	20 ng
*EF1α* NM_001402.5	F CCCCGACACAGTAGCATTTG R TGACTTTCCATCCCTTGAACC	98 bp	56 °C	25 (37)	20 ng (10 ng)
*IL-6* NM_000600.4	F AACCTGAACCTTCCAAAGATGG R TCTGGCTTGTTCCTCACTACT	159 bp	58 °C	30 (40)	20 ng (10 ng)
*MMP9* NM_004994.3	F ATGAGCCTCTGGCAGCCCCT R CCGTGCTCCGCGACACCAAA	527 bp	60 °C	35	20 ng
*RUNX2* NM_001024630.4	F CTGTGGTTACTGTCATGGCG R GGGAGGATTTGTGAAGACGGT	170 bp	60 °C	35	20 ng
*SOX9* NM_000346.3	F GAAGGACCACCCGGATTACA R GCCTTGAAGATGGCGTTGG	120 bp	60 °C	35	20 ng
*Tie2* NM_000459.5	F GGTCAAGCAACCCAGCCTTTTC R CAGGTCATTCCAGCAGAGCCAA	121 bp	64 °C	40 (37)	20 ng (10 ng)
*VEGFA* NM_001204384.1	F CTACCTCCACCATGCCAAGT R GCAGTAGCTGCGCTGATAGA	109 bp	60 °C	30 (40)	20 ng (10 ng)
*VEGFR2* NM_002253.2	F CAGGGGACAGAGGGACTTG R GAGGCCATCGCTGCACTCA	91 bp	60 °C	35 (40)	20 ng (10 ng)

^1^ With F: forward and R: reverse; ^2^ T_An_: Annealing Temperature; ^3^ n_cycles_: number of cycles; ^4^ m_CDNA_: amount of cDNA.

## Data Availability

The data used to support the findings of this study can be made available by the corresponding author upon request.

## Supplementary Materials

The following supporting information can be downloaded at: https://www.mdpi.com/article/10.3390/foods12152960/s1, Supplementary Figure S1: Graphic representation of experimental setup of the co-culture; Supplementary Figure S2: Establishment of co-culture conditions for in vitro fracture hematomas and HUVECs; Supplementary Figure S3: Total protein content of HUVECs stimulated with different concentrations of GE and MBE; Supplementary Figure S4: Cytokine secretion determined after 48 h in whole cell culture supernatants.

## Abbreviations

(RT–) PCR	(Reverse transcription–) Polymerase chain reaction
A/A	Antibiotic/Antimycotic
ALP	Alkaline Phosphatase
Angpt	Angiopoietin
ATP	Adenosine triphosphate
AUC	Area under the curve
CCL	C-C motif chemokine
CD (31)	Cluster of differentiation (31)
cDNA	Copy DNA
Col I	Collagen I
CSE	Cigarette smoke extract
Dp-3-g	Delphinidin-3-glucoside
EBM-2	Endothelial Cell Growth Basal Medium 2
EF1α	Eukaryotic translation elongation factor 1 alpha 1
EGF	Epidermal growth factor
EGM2-MV	Endothelial Cell Growth Medium 2—Microvascular
ELISA	Enzyme-linked-immunosorbent-assay
FCS	Fetal calf serum
FGF	Fibroblast growth factor
GE	*Panax ginseng* extract
HUVECs	Human umbilical vein endothelial cells
IGF	Insulin-like growth factor
IL	Interleukin
LDH	Lactate dehydrogenase
MBE	Maqui berry extract
MEM-α	Minimal Essential Medium Alpha
MSC	Mesenchymal stem cell
N	Non-smokers condition
P/S	Penicillin/Streptomycin
*P. ginseng*	*Panax ginseng*
PBS	Phosphate buffer saline
PDGF	Platelet-derived growth factor
PIGF	Platelet-induced growth factor
pNp	4-Nitrophenol
pNpp	4-Nitrophenyl phosphate
pNRF2	Phosphorylated nuclear factor erythroid 2—related factor 2
RAU	Relative absorbance units
RFU	Relative fluorescence units
RLU	Relative luminescence units
ROS	Reactive oxygen species
RUNX2	Runt-related transcription factor 2
RT-PCR	Reverse transcription polymerase chain reaction
S	Smokers conditions
S + GE	Smokers conditions with 10 µg/mL GE
S + MBE	Smokers conditions with 1 µg/mL MBE
SEM	Standard error of the mean
SRB	Sulforhodamine B
SOX9	SRY-Box Transcription Factor 9
TGF-β	Transforming growth factor beta
TNF-α	Tumor necrosis factor-alpha
VEGF	Vascular endothelial growth factor
VEGFR	Vascular endothelial growth factor receptor
