# Supplementary material for: Herbal Extracts of Ginseng and Maqui Berry Show Only Minimal Effects on an In Vitro Model of Early Fracture Repair of Smokers
Source: Foods. 2023 Aug 4;12(15):2960. doi: 10.3390/foods12152960 (PMC10419284; doi:10.3390/foods12152960)

Supplementary Figure S1. Graphic representation of experimental setup of the co-culture.

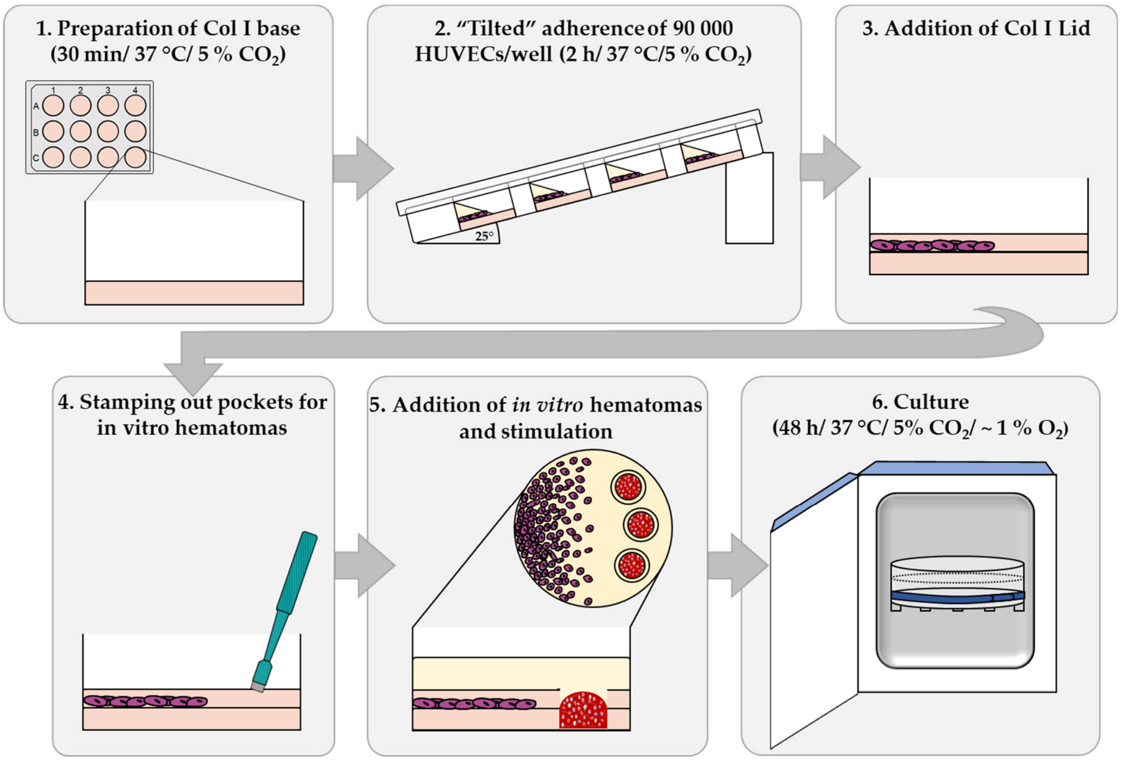

Supplementary Figure S2. Establishment of co-culture conditions for the in vitro fracture hematomas and HUVECs.

For establishing appropriate media conditions for co-culturing the in vitro fracture hematomas and HUVECs, different ratios of the respective medium MEM-a and EGM2-MV were screened in 25 % increments in regard to viability and culture specific markers for a total incubation time of 48 h. With (a) ATP content and LDH release of in vitro fracture hematomas. (b) ATP content and LDH release of HUVECs. (c) gene expression analysis of in vitro fracture hematomas for CCL2, RUNX2, SOX9 and VEGFA. (d) gene expression analysis of HUVECs for CD31, VEGF and VEGFR2. All data are shown as mean ± SEM. Viability data was collected in N=3, n=3. Gene expression data in N=3, n=2. For (a) and (c) statistics were made comparing to 0:100 only, for (b) and (d) statistics were made to 100:0 only. Levels of significance were defined as * $p < 0.05$, ** $p < 0.01$, and **** $p < 0.0001$.

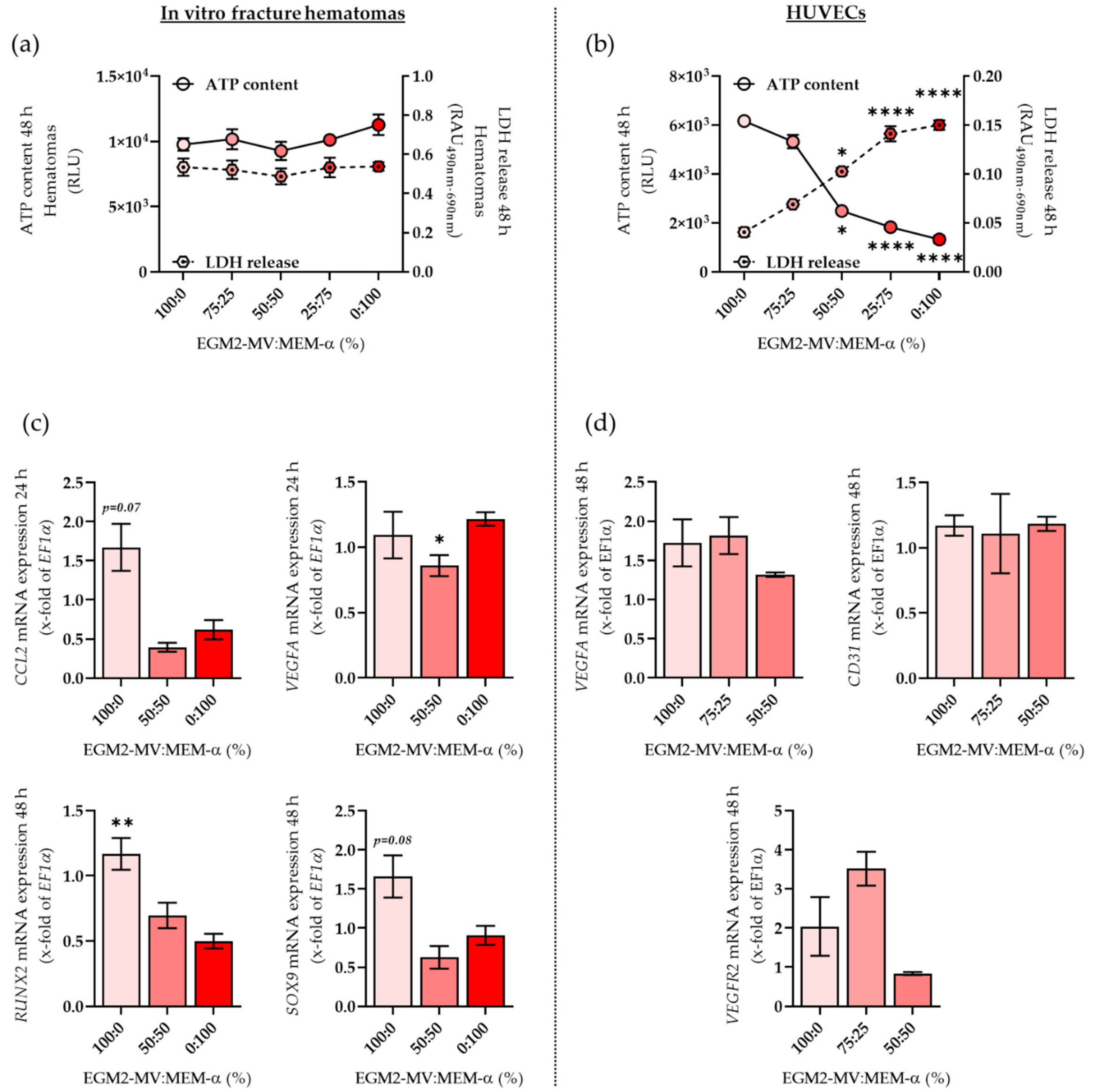

Supplementary Figure S3. Total protein content of HUVECs stimulated with different concentrations of GE and MBE.

Total protein content of HUVECs stimulated w/o or with 0.1, 1, 10, 50, or 100 µg/mL GE (left) or MBE (right) for 48 h in a hypoxic environment, was determined by SRB staining. All data are shown as Tuckey blots, whereas small black dots indicate outliers. Data were collected in N=4, n=3 and normalized to w/o stimulation. Control conditions w/o stimulation are indicated by the dotted line. Statistics were made in comparison to w/o only using non-parametric Kruskal-Wallis tests. Levels of significance were defined as * $p < 0.05$.

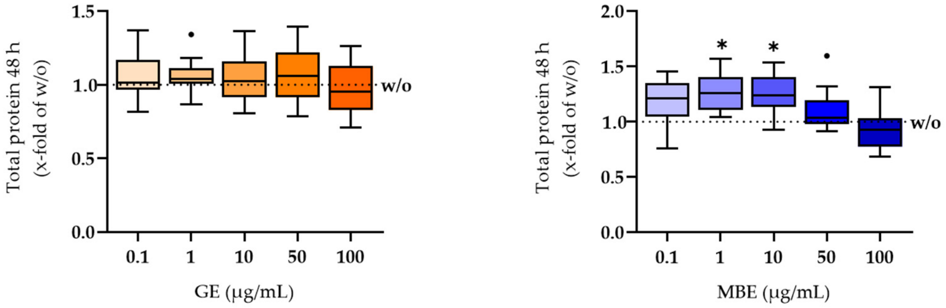

Supplementary Figure S4. Cytokine secretion determined after 48 h in whole cell culture supernatants.

Secretion of TNF-a, IL-6, and CCL2 in co-culture supernatants after 48 h of incubation determined by ELISA. N=5, n=3. Data are shown as Tuckey blots. Outliers are represented as small black dots.

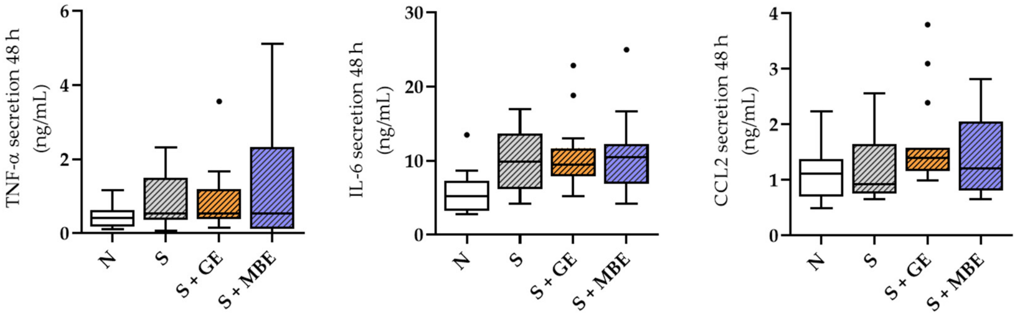

Supplement: Supplementary file 1 [file foods-12-02960-s001.zip › foods-2526738-supplementary.pdf]
